# Integrating pragmatic and implementation science randomized clinical trial approaches: a PRagmatic Explanatory Continuum Indicator Summary-2 (PRECIS-2) analysis

**DOI:** 10.1186/s13063-023-07313-0

**Published:** 2023-04-21

**Authors:** Douglas Zatzick, Lawrence Palinkas, David A. Chambers, Lauren Whiteside, Kathleen Moloney, Allison Engstrom, Laura Prater, Joan Russo, Jin Wang, Khadija Abu, Matt Iles-Shih, Eileen Bulger

**Affiliations:** 1 Department of Psychiatry and Behavioral Sciences, University of Washington School of Medicine, Seattle, USA; 2 Department of Children, Youth, and Families, California School of Social Work, University of Southern, Los Angeles, CA USA; 3 Implementation Science, Division of Cancer Control and Population Sciences, National Cancer Institute, Rockville, MD USA; 4 Department of Emergency Medicine, University of Washington School of Medicine, Seattle, USA; 5 Department of Surgery, University of Washington School of Medicine, Seattle, USA

**Keywords:** Pragmatic clinical trials, Implementation science, Health care systems, PRECIS-2, PRECIS-2-PS, Policy, Alcohol screening and brief intervention, Posttraumatic stress disorder

## Abstract

**Background:**

Over the past two decades, pragmatic and implementation science clinical trial research methods have advanced substantially. Pragmatic and implementation studies have natural areas of overlap, particularly relating to the goal of using clinical trial data to leverage health care system policy changes. Few investigations have addressed pragmatic and implementation science randomized trial methods development while also considering policy impact.

**Methods:**

The investigation used the PRagmatic Explanatory Continuum Indicator Summary-2 (PRECIS-2) and PRECIS-2-Provider Strategies (PRECIS-2-PS) tools to evaluate the design of two multisite randomized clinical trials that targeted patient-level effectiveness outcomes, provider-level practice changes and health care system policy. Seven raters received PRECIS-2 training and applied the tools in the coding of the two trials. Descriptive statistics were produced for both trials, and PRECIS-2 wheel diagrams were constructed. Interrater agreement was assessed with the Intraclass Correlation (ICC) and Kappa statistics. The Rapid Assessment Procedure Informed Clinical Ethnography (RAPICE) qualitative approach was applied to understanding integrative themes derived from the PRECIS-2 ratings and an end-of-study policy summit.

**Results:**

The ICCs for the composite ratings across the patient and provider-focused PRECIS-2 domains ranged from 0.77 to 0.87, and the Kappa values ranged from 0.25 to 0.37, reflecting overall fair-to-good interrater agreement for both trials. All four PRECIS-2 wheels were rated more pragmatic than explanatory, with composite mean and median scores ≥ 4. Across trials, the primary intent-to-treat analysis domain was consistently rated most pragmatic (mean = 5.0, SD = 0), while the follow-up/data collection domain was rated most explanatory (mean range = 3.14–3.43, SD range = 0.49–0.69). RAPICE field notes identified themes related to potential PRECIS-2 training improvements, as well as policy themes related to using trial data to inform US trauma care system practice change; the policy themes were not captured by the PRECIS-2 ratings.

**Conclusions:**

The investigation documents that the PRECIS-2 and PRECIS-2-PS can be simultaneously used to feasibly and reliably characterize clinical trials with patient and provider-level targets. The integration of pragmatic and implementation science clinical trial research methods can be furthered by using common metrics such as the PRECIS-2 and PRECIS-2-PS. Future study could focus on clinical trial policy research methods development.

**Trial registration:**

DO-SBIS ClinicalTrials.gov NCT00607620. registered on January 29, 2008. TSOS ClinicalTrials.gov NCT02655354, registered on July 27, 2015.

## Contributions to the literature

- The investigation documents that the PRECIS-2 and PRECIS-2-PS can be simultaneously used to feasibly and reliably rate randomized clinical trials with both patient and provider-level targets.
- The combined use of the PRECIS-2 and PRECIS-2-PS tools may provide clarity for clinical trial planning and grant submission and review, and enhance the quality of clinical trial protocol and main outcome manuscript publications.
- Future clinical trials research methods development could focus on continuum indicators/checklists that assess the extent to which clinical trials successfully catalyze health care system practice changes that are linked to pre-specified policy targets.

## Background

Over the past decade, both pragmatic and implementation science clinical trial research methods have advanced substantially [1–8]. The US National Institutes of Health recently released an application request that jointly solicits proposals for embedded health care system pragmatic and implementation randomized controlled trials [9]. Pragmatic trials are characterized by a primary purpose of understanding the effects of an intervention under usual practice conditions [9]. Implementation studies aim to understand the behavior of practitioners and others working within health care systems as key influences on the adoption, implementation, and sustainability of interventions and practice guidelines, while implementation trials test strategies designed to improve use of and/or fidelity to effective interventions. Per the application request, pragmatic and implementation clinical trial results should optimally inform policy makers and other key stakeholders [9].

Pragmatic and implementation randomized clinical trials have natural areas of overlap. For example, multisite pragmatic and implementation trials grapple with similar regulatory issues [10–12]. Pragmatic and implementation trials may be designed to target both individual patient effectiveness and provider-level practice change outcomes [5, 7, 8, 13–15]. Also, cross-cutting commentary has encouraged the integration of policy targets into pragmatic and implementation clinical trial designs as an approach to accelerating health care system practice changes derived from clinical trial results [5, 14, 16–21]. Methodologic integration across pragmatic and implementation trials has the potential to provide clarity to clinical trial planning and grant submission and review and to improve the quality of clinical trial protocol and main outcome manuscript publications [9, 22]. A literature review, however, revealed few investigations that have directly addressed applied methodologic integration across the two fields while also incorporating policy perspectives.

Prior investigations straddling pragmatic and implementation clinical trial approaches have focused on the Pragmatic-Explanatory Continuum Indicator Summary (PRECIS), a tool for rating clinical trials on the pragmatic-explanatory spectrum [2, 3, 22–26]. The PRECIS tool allows clinical investigators, grant reviewers, journal editors, and others to assess multiple patient-level randomized clinical trial domains across the explanatory-pragmatic trial continuum. A pioneering investigation from a National Institute of Health, Health Care System Research Collaboratory investigative team, used the PRECIS-2 tool to evaluate and compare five pragmatic trials [24]. Along with quantitative PRECIS-2 ratings of the five trials, the investigation included notational comments regarding the feasibility of using the PRECIS-2 tool.

More recently, the PRECIS-2-Provider Strategies (PS) tool has been developed with the aim of better characterizing trials where participants are health care providers [3, 22]. The tool is intended to clarify the rating of trials that are targeting provider-level rather than patient-level outcomes [22]. A literature review revealed no published investigations that have simultaneously used the PRECIS-2 and PRECIS-2-PS tools to rate trials with both patient- and provider-level targets.

Over the past decade, members of the investigative team have developed and rolled out two multi-site cluster randomized clinical trials in US trauma care systems, the Disseminating Organizational Screening and Brief Intervention Services (DO-SBIS) and the Trauma Survivors Outcomes and Support (TSOS) investigations [27–30]. The DO-SBIS and TSOS trials trained a broad spectrum of front-line trauma center providers in behavioral interventions with the aim of improving the quality of mental health and substance use screening and intervention procedures and patient-level outcomes. Also, the DO-SBIS and TSOS trials aimed to use clinical trial results to impact national trauma care system policy. The American College of Surgeons Committee on Trauma (ACS/COT) has the capacity to invoke health care system-level regulatory policies that are guided by randomized clinical trial results. [14, 31–33]. Both the DO-SBIS and TSOS trials included as a core design element, a multistep planning process targeting national trauma care system policy impact, that included an end-of-study policy summit [27–30]. The policy summit agendas included a review of the pragmatic trial results augmented by stakeholder participant commentary. Participating stakeholders included clinician scientists, trauma surgical policy makers, patient injury survivors, frontline trauma center providers, and grant/contract program personnel.

The current investigation builds upon and extends these prior investigations by working to further the methodologic integration of pragmatic and implementation science clinical trial approaches while simultaneously incorporating policy research perspectives. The investigation used the PRECIS-2 and PRECIS-2-PS tools to rate patient and provider-level characteristics of the DO-SBIS and TSOS trials. The investigation examined whether both tools could be simultaneously used to feasibly and reliably rate the two trials. The study employed the Rapid Assessment Procedure Informed Clinical Ethnography (RAPICE) qualitative approach to systematically document observations regarding the feasibility of using the PRECIS-2 and PRECIS-2-PS to rate the trials. RAPICE was also used to make clinical trial-related policy observations. The RAPICE observations sought to identify PRECIS-2 and PRECIS-2-PS domains that overlapped with or were potentially impacted by College policy. Given that the original intent of the PRECIS tools did not include a policy focus, RAPICE also aimed to augment the PRECIS coding with policy relevant observations.

## Methods

### Design overview

The investigation combined quantitative patient-focused PRECIS-2 and provider-focused PRECIS-2-PS tool ratings of the DO-SBIS and TSOS clinical trials with the qualitative RAPICE approach. The TSOS and DO-SBIS clinical trials have been previously described in detail [27–30]. Briefly, conducted between 2007 and 2013, the DO-SBIS trial randomized 20 US level I trauma centers to the ACS alcohol screening and intervention mandate as usual care control (*n* = 10) versus study team alcohol screening and brief intervention training (*n* = 10) conditions. A policy summit was scheduled during the final portion of the DO-SBIS study. The TSOS trial was built upon methods developed in the DO-SBIS trial and was conducted between 2015 and 2020. The TSOS trial aimed to test the effectiveness of evidence-based screening and interventions for Post-traumatic stress disorder (PTSD) and mental health and substance use comorbidities at 25-level US Level I trauma centers nationwide. As with the DO-SBIS trial, an end-of-study ACS policy summit was proactively planned for the final years of the TSOS trial.

All DO-SBIS procedures were approved by the University of Washington Institutional Review Board prior to the initiation of the investigation. All TSOS procedures were approved by the Western Institutional Review Board before the initiation of patient recruitment. The RAPICE study procedures were approved by the University of Washington Institutional Review Board.

## PRECIS rating procedures

Previously published PRECIS-2 and PRECIS-2-PS manuscripts and tool descriptions informed the rating procedures [3, 22, 24]. The original patient-focused PRECIS-2 evaluates trials on the pragmatic-explanatory continuum across nine domains, including eligibility, recruitment, setting, organization, flexibility-delivery, flexibility-adherence, follow-up, primary outcome, and primary analysis. The PRECIS-2-PS expands the coding to incorporate provider-focused implementation domains that include eligibility, recruitment, setting, implementation resources, flexibility of provider strategies, flexibility of intervention, data collection, primary outcome, and primary analysis. Each domain is scored on a 5-point Likert scale that ranges from 1—very explanatory (center of the wheel) to 5—very pragmatic (rim of the wheel).

The PRECIS raters included seven study team members at the University of Washington’s Harborview Medical Center (DZ, LW, KM, AE, LP, JR, JW). The raters volunteered to participate on the project and were recruited based on their availability and interest. The raters had varying familiarity with the DO-SBIS and TSOS trials. None of the raters had previously participated in PRECIS-2 coding exercises.

All raters received PRECIS-2 and PRECIS-2-PS coding training tailored to the acute care medical context in which the DO-SBIS and TSOS trials were conducted. The training consisted of an initial PowerPoint zoom call orientation with the lead investigator (DZ). The initial presentation reviewed the original PRECIS-2 and PRECIS-2-PS publications as well as previous manuscripts documenting PRECIS coding efforts. The raters were instructed to derive their PRECIS-2 and PRECIS-2-PS ratings from a review of the DO-SBIS and TSOS trials study protocol and main outcome manuscripts [27–30].

The initial coding effort raised questions for multiple raters regarding PRECIS-2 explanatory-pragmatic continuum domain scaling. A recalibration slide set that added example figures and published manuscripts to better articulate the PRECIS-2 explanatory-pragmatic continuum scaling was therefore developed [3, 22, 34–37]. The slide set was sent via email to the raters, and all raters were given the opportunity to discuss the recalibration training slide set with the lead investigator. Final data presented in the tables represent post-recalibration training scoring.

## Quantitative data analyses

Consistent with other studies using the PRECIS, rater means, standard deviations, medians, and ranges were produced for each of the nine patient and provider domains for both trials [24]. The mean scores for each domain were plotted on the PRECIS-2 and PRECIS-2-PS wheel diagrams. Interrater agreement was measured with the intraclass correlation coefficient (ICC) and the Kappa statistics.

## RAPICE data collection and analysis

The RAPICE qualitative approach has been described in detail in prior publications [13, 38, 39]. Core features of the RAPICE approach include the formation of an interdisciplinary team, use of multiple data sources, iterative data collection, analysis in real-time, and rapid completion of the project. RAPICE is designed to be adaptable to diverse real world research contexts and embeds participant observation within clinical research protocols. Investigators conducting clinical research are trained in RAPICE methods, including the logging and analysis of field notes. In the current study, RAPICE field notes were taken during PRECIS-2 training by the lead investigator (DZ); the field notes incorporated general observations of the training content, questions asked by the raters, and modifications required of the training materials to enhance feasibility of use of the tools. Also, RAPICE field notes were taken during the TSOS end-of-study policy summit by the investigation’s expert mixed methods co-investigator (LP).

Data analyses in RAPICE incorporate two different data analysis procedures. The immersion/crystallization style was used to provide interpretations of the data by both DZ and LP and consisted of a prolonged immersion into and experience with the text and then emerging, after concerned reflection, with an intuitive crystallization of the text [40]. The more time-intensive editing style utilized a methodology of focused thematic analysis [41]. Field notes were coded by LP to condense the data into analyzable units. Segments of text ranging from a phrase to several paragraphs were assigned codes based on a priori or emergent themes (also known as open coding) [13]. Following the open coding, codes were assigned to describe connections between and within categories (also known as axial coding). Codes were then combined into groups to generate themes through a process of constant comparison.

## Results

Descriptive characteristics of the DO-SBIS and TSOS trials are presented in Table 1. Both trials were cluster randomized and included ≥ 20 trauma center sites. The trials each recruited ≥ 600 patients and trained a full spectrum of front-line trauma center providers in the respective intervention approaches. Both trials include a planned end-of-study policy summit.

**Table 1:** Characteristics of the DO-SBIS and TSOS randomized clinical trials

Trial characteristic	DO-SBIS	TSOS
Number of US trauma center sites	20	25
Clinical trial design	Parallel group cluster randomized	Stepped wedge cluster randomized
Patient recruitment	May 2009–September 2011	January 2016–November 2018
Number of patients	878	635
Research question	Can a brief acute care intervention reduce alcohol use problems over the course of the year after injury?	Can stepped collaborative care reduce the symptoms of Posttraumatic stress disorder and related comorbidity?
Intervention	Alcohol screening and brief motivational interview intervention	Stepped collaborative care
Control	Mandate as usual alcohol screening and brief intervention	Usual care control
Provider groups receiving intervention training	Social work, nursing, clinical psychology	Social work, nursing, clinical psychology, physicians
US trauma care system policy directive targeted	Alcohol screening and intervention	Posttraumatic stress disorder and related comorbidity screening, intervention, and referral
End-of-study ACS/COT policy summit	Yes	Yes

*DO-SBIS* Disseminating Organizational Screening and Brief Intervention Services, *TSOS* Trauma Survivors Outcomes and Support, *ACS/COT* American College of Surgeons Committee on Trauma

**Fig. 1:**
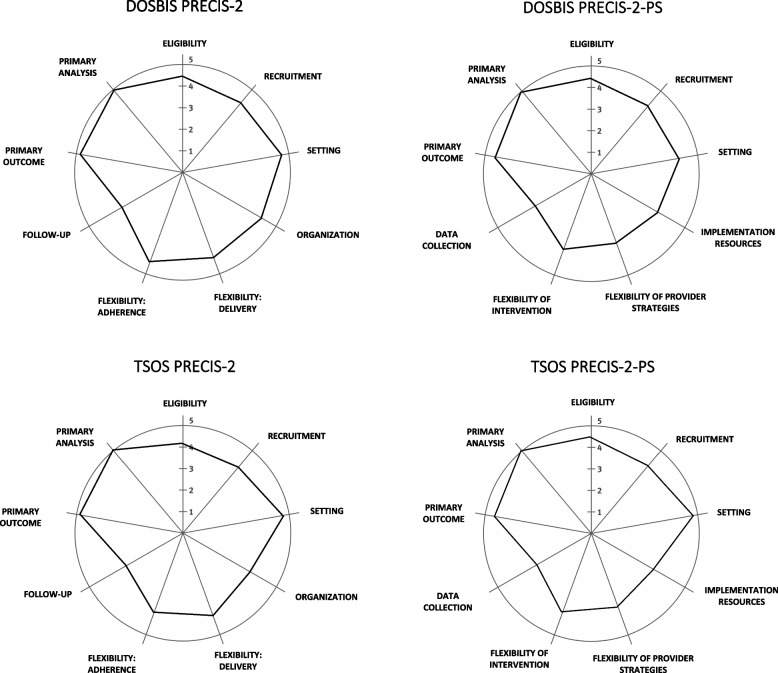
Disseminating Organizational Screening and Brief Intervention Services (DO-SBIS) and Trauma Survivors Outcomes and Support (TSOS), PRagmatic Explanatory Continuum Indicator Summary-2 (PRECIS-2) and PRagmatic Explanatory Continuum Indicator Summary-2 -Provider Strategies (PRECIS-2-PS), wheel diagrams

The PRECIS-2 and PRECIS-2-PS wheels are displayed for both the TSOS and DO-SBIS trials in Fig. 1. All four PRECIS-2 wheels were rated to be more pragmatic than explanatory, with overall composite mean and median scores ≥ 4 (Table 2).

**Table 2:** PRECIS-2 and PRECIS-2-PS ratings for the TSOS and DO-SBIS trials

PRECIS-2 domain	TSOS patients Mean (SD)	DO-SBIS patients Mean (SD)	PRECIS-2-PS domain	TSOS providers Mean (SD)	DO-SBIS providers Mean (SD)
1. Eligibility	4.14 (0.38)	4.43 (0.53)	1. Eligibility	4.43 (0.53)	4.43 (0.53)
2. Recruitment	4.00 (0.58)	4.29 (0.49)	2. Recruitment	4.14 (0.69)	4.14 (0.38)
3. Setting	4.71 (0.49)	4.71 (0.49)	3. Setting	4.86 (0.38)	4.29 (1.11)
4. Organization	3.79 (0.70)	4.29 (0.49)	4. Implementation Resources	3.43 (0.53)	3.71 (0.76)
5. Flexibility-delivery	4.14 (0.69)	4.29 (0.49)	5. Flexibility-provider	3.71 (0.76)	3.57 (1.13)
6. Flexibility-adherence	4.00 (1.15)	4.43 (0.53)	6. Flexibility-intervention	4.00 (0.82)	3.86 (0.69)
7. Follow-up	3.29 (0.49)	3.43 (0.53)	7. Data collection	3.14 (0.69)	3.14 (0.69)
8. Primary outcome	4.86 (0.38)	4.86 (0.38)	8. Primary outcome	4.57 (0.79)	4.57 (0.79)
9. Primary analysis	5.00 (0.00)	5.00 (0.00)	9. Primary analysis	5.00 (0.00)	5.00 (0.00)
Mean (SD)	4.21 (0.55)	4.41 (0.45)	Mean (SD)	4.14 (0.63)	4.08 (0.57)
Median (range)	4.14 (1.71)	4.43 (1.57)	Median (range)	4.14 (1.86)	4.14 (1.86)
ICC	0.85 (0.62–0.96)	0.87 (0.68–0.97)	ICC	0.87 (0.68–0.97)	0.77 (0.44–0.94)
Kappa	0.29 (0.14–0.43)	0.33 (0.15–0.50)	Kappa	0.25 (0.10–0.40)	0.37 (0.22–0.52)

*DO-SBIS* Disseminating Organizational Screening and Brief Intervention Services, *TSOS* Trauma Survivors Outcomes and Support

Across trials, ICCs ranged from 0.77 to 0.87, and Kappa values ranged from 0.25 to 0.37 (Table 2). For both trials, the primary analysis domain was consistently rated most pragmatic (mean = 5.0, SD = 0), while the follow-up/data collection domain was rated most explanatory (mean range = 3.14–3.43, SD range = 0.49–0.69). The greatest rater variability was observed for the TSOS patient flexibility (adherence, mean = 4.0, SD = 1.15) and DO-SBIS provider setting (mean = 4.29, SD = 1.11) and flexibility of provider-focused strategies (mean = 3.57, SD = 1.13) domains.

Analysis of the PRECIS-2 training and policy summit RAPICE field notes revealed two broad themes: (1) challenges to the feasible use of PRECIS and (2) benefits of combined use of PRECIS and RAPICE. Two challenges to the use of PRECIS-2 were identified, challenges associated with training raters and challenges associated with operationalization of the measure. Multiple raters noted a lack of attention to detailed domain scaling in the initial coding training (Table 3). Two raters noted a lack of detail in the protocol and main outcome papers regarding the selection and training of providers. Other themes identified by individual raters included the need for enhanced definitions of terminology required for domain coding and rater variation in knowledge of trial details.

**Table 3:** Rapid Assessment Procedure Informed Clinical Ethnography (RAPICE) observations and implications

Theme	Observation	Implications/relevance	Data source
PRECIS training challenges	Multiple raters (3) noted that the scaling of individual domains had not been adequately detailed in the initial training	A recalibration training slide set was developed that added example manuscripts and figures designed to better articulate the PRECIS-2 explanatory-pragmatic continuum	PRECIS-2 training field notes
	Multiple raters (2) noted the lack of detail in the protocol and main outcome papers regarding the selection and training of providers	Importance of reviewing source materials for comprehensive reporting of characteristics necessary to rate multiple domains for patient and provider PRECIS-2 diagrams	PRECIS-2 training field notes
PRECIS operationalization challenges	PRECIS-2-PS domain 4 is challenging to code without a more detailed knowledge of the meaning of provider-focused “strategies” (for example, are these to be more formally considered as distinct implementation strategies?)	The clarity of domain descriptions could be improved by use of terms free of alternative meanings/implications	PRECIS-2 training field notes
PRECIS/RAPICE convergence	The follow-up/data collection domain was rated to be more explanatory as the procedures involved (e.g., standardized patient interviews in the DO-SBIS study) were not grounded in routine patient care	Descriptive observations derived from field notes align with quantitative PRECIS ratings	PRECIS-2 training field notes
	One rater with extensive experience across trials asked very detailed comparative questions regarding DO-SBIS and TSOS	Differences in rater knowledge of individual trials can lead to interrater variability	PRECIS-2 training field notes
PRECIS/RAPICE complementarity	Not mentioned in the DO-SBIS protocol or main outcome paper is the observation that a national alcohol screening and intervention requirement could limit the patient’s flexibility of delivery domain	Important unappreciated impact of national policy on PRECIS-2 ratings	PRECIS-2 training field notes
PRECIS/RAPICE complementarity	1. Providers and ACS/COT have different perceptions of importance of screening guidelines 2. Responsibility for implementation 3. New ACS/COT screening and referral requirement for psychological distress 4. Evidence required for successful implementation comes from researchers, former patients and practitioners, all of whom must be engaged	The PRECIS-2 and PRECIS-2-PS tools currently lack policy-relevant domains addressing sustainable implementation of practices derived from pragmatic trial procedures and results. Questions arise as to whether an explanatory-pragmatic continuum/domain could be developed that calibrated the use of policy levers or other means to catalyze the translation of clinical trial results into real world practice changes	Trauma Survivors Outcomes & Support policy summit field notes

The benefits of the mixed methods use of both PRECIS-2 and RAPICE were also twofold. One benefit was convergence of findings from both methods, illustrated in the similar emphasis related to the rationale for rating some domains as more explanatory. Another benefit was complementarity due to the availability of information obtained from one method but not the other. This was illustrated in the underappreciated restrictive impact of ACS/COT national policy, specifically on the DO-SBIS patient, flexibility of delivery, domain, and the optimal approaches to the translation of clinical trial results into ACS/COT standards and associated sustainable US trauma care system practice changes revealed in the RAPICE analysis of policy summit field notes. Analysis of the RAPICE policy summit observations information revealed four subthemes. The first subtheme was the difference in perception of ACS/COT recommendations by trauma surgeons and the ACS/COT. The second subtheme pertained to the distinction between a top-down versus a bottom-up responsibility for implementation. The third subtheme pertained to discussions of the new psychological symptoms screening and referral requirement for US Trauma Care Systems. The fourth subtheme pertained to the evidence required for successful implementation derived from different stakeholder groups.

## Discussion

The current study builds upon and extends prior investigations that have straddled implementation science and pragmatic clinical trial approaches [2, 3, 22, 23]. The study, for the first time, used the PRECIS-2 and PRECIS-2-PS tools to characterize two health care system patient- and provider-focused randomized trials that incorporated ACS/COT policy targets. Overall, coders rated both trials to be quite pragmatic, with composite mean and median PRECIS-2 and PRECIS-2-PS scores of ≥ 4. Although the DO-SBIS clinical trial temporally preceded the TSOS trial, the two investigations had multiple design similarities. Both trials trained a broad spectrum of providers at 20 or more trauma center sites to implement behavioral interventions with hundreds of patients. Both trials included planned intent-to-treat main outcome analysis and research follow-up assessments. Regarding complimentary aspects of the PRECIS-2 and PRECIS-2-PS wheels, coding with the two tools identified the similarities between the trials. Across trials and PRECIS-2 wheels, the intent-to-treat data analysis domain was consistently rated most pragmatic, while the follow-up/data collection domain was consistently rated most explanatory.

The investigation found fair-to-good interrater reliability on the PRECIS-2 and PRECIS-2-PS as assessed by the ICC and Kappa statistics [42, 43]. The PRECIS-2-PS was developed to capture domains relevant to provider-focused trials [22]. Other PRECIS investigations have characterized multiple pragmatic trials across patient-focused domains without detailed attention to provider characteristics [24–26]. The current investigation furthers the integration of pragmatic trial and implementation science methods by documenting that the PRECIS-2 and PRECIS-2-PS can be feasibly and reliably applied to characterize trials focused on patient- and provider-level outcomes. These findings suggest that the combined use of the two tools could enhance conceptual and methodological clarity in the area of clinical trial planning by facilitating an improved match between patient and provider-level design decisions and overall trial purpose [22]. The combined use of the two tools may also enhance conceptual and methodological clarity for clinical trial grant submissions and review, and clinical trial protocol and main outcome manuscript publications [9, 22].

Commentary spanning pragmatic and implementation science clinical trial approaches suggests that policy integration could accelerate the research-to-practice translation; the recent NIH combined pragmatic and implementation trial announcement emphasized the importance of clinical trial policy relevance [5, 9, 14, 16–21]. The ACS/COT has the capacity to invoke health care system level policy implementation strategies, including regulatory requirements linked to national trauma center verification site visits [44]. Both the DO-SBIS and TSOS trials directly targeted the advancement of national trauma care system regulatory guidelines and included a multistep policy planning process. The letters of support submitted in the original grant applications obtained up-front commitments from key US trauma care system policy stakeholders. A planned end-of-study ACS/COT summit that convened stakeholders for a review of policy-relevant clinical trial findings was a central element of both trials. The presentation of policy-relevant findings derived from the trials was planned to occur within a single grant cycle, effectively reducing the time delay between the production of clinical trial research results and widespread health care system practice changes [6, 14, 16, 21].

A common pragmatic trial investigative approach has been to work inductively from individual trials to identify potentially integrative methods [13, 14, 45]. One goal of the current investigation was to use RAPICE observations to identify PRECIS domains that overlapped with or were impacted by College policy. For example, RAPICE field observations noted that College requirements for specific alcohol screening and brief intervention procedures had the potential to delimit trauma center usual care, thus impacting the PRECIS-2-PS “flexibility of intervention” domain. In addition, a key study RAPICE observation was that the PRECIS-2 and PRECIS-2-PS domain coding did not capture many themes related to catalyzing the research-to-practice translation through a multiphasic policy planning process. Other pragmatic trials have engaged health care system policy makers up-front in trial design and roll-out to expedite the translation of clinical trial results; these prior pragmatic trial efforts have focused both on the end-of-study implementation of novel practices found to be beneficial and the de-implementation of practices found to be redundant/without benefit to patients [45–48]. This initial constellation of policy-focused health care system randomized clinical trials could be seen to constitute examples of the emerging “learning health policy systems” construct [17].

Future research could refine the learning health policy systems construct as it relates to pragmatic and implementation randomized clinical trials. Additional investigation could productively focus on the development of continuum indicators/checklists that assess the extent to which clinical trials successfully catalyze health care system practice changes that are temporally linked to pre-specified policy targets. Elements for continuum/checklist ratings might include incorporation of policy levers up-front in clinical trial design, ratings of policy makers’ initial buy-in and sustained commitment to incorporating trial results, and whether the trial included an explicit mechanism for the health care system integration of findings, such as an end-of-study policy summit. Of note, such checklists could be derived from previously identified policy-relevant implementation domains [49]. Ultimately a key retrospective indicator would be whether findings were incorporated into health care system practice changes. One hypothesis associated with this scaling would be investigations that include greater up-front buy-in, linkage of trial results to health care system policy levers, and end-of-study review will demonstrate diminished research-to-practice translation timelines [14, 21]. As with the combined application of the PRECIS-2 and PRECIS-2-PS, the use of novel learning health policy systems indicators could advance the quality of clinical trial planning, grant submission, grant review, and protocol and main outcome manuscript publications.

The investigation also contributes to an evolving literature on PRECIS coder training. Some [23, 25], but not all [24], prior PRECIS studies have described feasible use of the PRECIS tools as well as reliable coder ratings. As with previously described PRECIS rater training efforts, in the current study, RAPICE observation suggested that coders required iterative modifications to the training procedures [24, 25]. In the current investigation, these efforts focused on clarifying the explanatory-pragmatic continuum for acute care medical intervention trials. Also, the protocol and main outcome manuscripts used by coders to retrospectively derive scores may not have included adequate information regarding the full spectrum of PRECIS-2 and PRECIS-2-PS domains.

The investigation has additional limitations. Raters were drawn from a single study team, and only two trials were rated. This approach may have contributed to decreased variability in ratings across PRECIS-2 and PRECIS-PS domains and pragmatic trials; raters did, however, have varying degrees of familiarity with the two trials. Also, the study team acknowledges that the PRECIS coding occurred at the conclusion of a muti-year investigation; prospective ratings may prove beneficial in future investigations. The training required iterative modifications. An alternative approach would have been to develop a lengthier, more rigorous training up-front. However, as with previous PRECIS training efforts, the study team’s overarching goal was to develop a lower-intensity, more generalizable training procedure that could require context-specific iterative modifications [25]. One strength of this approach is the potential broader application of the PRECIS-2 tools across raters with disparate research and/or clinical backgrounds.

Beyond these considerations, this investigation also contributes to an evolving literature on the ability of the RAPICE approach to systematically augment quantitative data collected as part of larger-scale health care system trials [13, 50, 51]. A growing literature describes the systematic incorporation of qualitative observation to augment quantitative findings of pragmatic and implementation clinical trials. Prior studies included notational comments regarding the feasibility of using the PRECIS-2 tool across multiple health care system pragmatic trials [24]. The RAPICE approach builds upon and expands prior investigation by more systematically documenting qualitative observations related both to feasibility of use of the PRECIS-2 and PRECIS-2-PS tools as well as systematically documenting qualitative observations related to policy summit proceedings.

## Conclusion

The investigation documents that the PRECIS-2 and PRECIS-2-PS can be simultaneously used to feasibly and reliably rate pragmatic clinical trial protocols. The quality of pragmatic and implementation clinical trial protocol design, grant review, and manuscripts submissions can be enhanced by the use of common metrics such as the PRECIS-2 and PRECIS-2-PS rating tools. Finally, to optimally address the research-to-practice gap, future integrative study could productively focus on the development of continuum indicators continuum indicators/checklists that assess the extent to which clinical trials successfully catalyze health care system practice changes that are linked to pre-specified policy targets.

## Data Availability

The data sets generated and/or analyzed during the current study are not publicly available but are available from the corresponding on reasonable request.

## Abbreviations

PRECIS-2: PRagmatic Explanatory Continuum /Indicator Summary-2
PRECIS-2-PS: PRECIS-2-Proivder Strategies
RAPICE: Rapid Assessment Procedure Informed Clinical Ethnography
DO-SBIS: Disseminating Organizational Screening and Brief Intervention Services
TSOS: Trauma Survivors Outcomes and Support
ICC: Intraclass correlation
RE-AIM: Reach, Effectiveness, Adoption, Implementation, Maintenance
ACS/COT: American College of Surgeons Committee on Trauma

## References

[1] Tunis SR, Stryer DB, Clancy CM (2003). Practical clinical trials: Increasing the value of clinical research for decision making in clinical and health policy. JAMA.

[2] Thorpe KE, Zwarenstein M, Oxman AD, Treweek S, Furberg CD, Altman DG (2009). A pragmatic-explanatory continuum indicator summary (PRECIS): a tool to help trial designers. J Clin Epidemiol.

[3] Loudon K, Treweek S, Sullivan F, Donnan P, Thorpe KE, Zwarenstein M (2015). The PRECIS-2 Tool: Designing trials that are fit for purpose. B MJ.

[4] Sox HC, Lewis RJ (2016). Pragmatic Trials: Practical Answers to "Real World" Questions. JAMA.

[5] Califf RM, Sugarman J (2015). Exploring the ethical and regulatory issues in pragmatic clinical trials. Clin Trials.

[6] Chambers D. Foreword. In: Ross C. Brownson GAC, Enola K. Proctor, editor. Dissemination and implementation research in health: Translating science to practice. 2nd ed. New York: Oxford University Press; 2018. p. ix-xv.

[7] Curran GM, Bauer M, Mittman B, Pyne JM, Stetler C (2012). Effectiveness-implementation hybrid designs: combining elements of clinical effectiveness and implementation research to enhance public health impact. Med Care.

[8] Landes SJ, McBain SA, Curran GM. An introduction to effectiveness-implementation hybrid designs. Psychiatry Res. 2019;280:112513. 10.1016/j.psychres.2019.112513. 10.1016/j.psychres.2019.112513 PMC6779135 31434011

[9] National Institute of Health (NIH) Healthcare Systems Research Collaboratory. Pragmatic and Implementation Trials of Embedded Interventions (Funding Opportunity Announcement Number: RFA-AT-22–001). 2022.

[10] Fiscella K, Sanders M, Holder T, Carroll JK, Luque A, Cassells A (2020). The role of data and safety monitoring boards in implementation trials: When are they justified?. J Clin Transl Sci.

[11] Ellenberg SS, Culbertson R, Gillen DL, Goodman S, Schrandt S, Zirkle M (2015). Data monitoring committees for pragmatic clinical trials. Clin Trials.

[12] Roberts MK, Fisher DM, Parker LE, Darnell D, Sugarman J, Carrithers J (2020). Ethical and regulatory concerns in pragmatic clinical trial monitoring and oversight. Ethics Human Res.

[13] Palinkas LA, Zatzick D (2019). Rapid assessment procedure informed clinical ethnography (RAPICE) in pragmatic clinical trials of mental health services implementation: Methods and applied case study. Adm Policy Ment Health.

[14] Zatzick D, Moloney K, Palinkas L, Thomas P, Anderson K, Whiteside L (2021). Catalyzing the translation of patient-centered research into United States trauma care systems: a case example. Med Care.

[15] Tuzzio L, Larson EB, Chambers DA, Coronado GD, Curtis LH, Weber WJ, et al. Pragmatic clinical trials offer unique opportunities for disseminating, implementing, and sustaining evidence-based practices into clinical care: proceedings of a workshop. Healthcare (Amsterdam, Netherlands). 2019;7(1):51–7. 10.1016/j.hjdsi.2018.12.003. 10.1016/j.hjdsi.2018.12.003 PMC6557660 30594497

[16] Balas EA, Boren SA. Managing clinical knowledge for health care improvement. In: J B, AT M, editors. Yearbook of Medical Informatics 2000: Patient-Centered Systems. Stuttgart: Schattauer Verlagsgesellschaft; 2000. 27699347

[17] Oh A, Abazeed A, Chambers DA. Policy implementation science to advance population health: the potential for learning health policy systems. Front Public Health. 2021;9:681602. 10.3389/fpubh.2021.681602. 10.3389/fpubh.2021.681602 PMC8247928 34222180

[18] Hoagwood KE, Purtle J, Spandorfer J, Peth-Pierce R, Horwitz SM (2020). Aligning dissemination and implementation science with health policies to improve children’s mental health. Am Psychol.

[19] Purtle J, Marzalik JS, Halfond RW, Bufka LF, Teachman BA, Aarons GA (2020). Toward the data-driven dissemination of findings from psychological science. Am Psychol.

[20] Purtle J, Nelson KL, Counts NZ, Yudell M (2020). Population-based approaches to mental health: history, strategies, and evidence. Annu Rev Public Health.

[21] Beidas RS, Dorsey S, Lewis CC, Lyon AR, Powell BJ, Purtle J (2022). Promises and pitfalls in implementation science from the perspective of US-based researchers: learning from a pre-mortem. Implement Sci.

[22] Norton WE, Loudon K, Chambers DA, Zwarenstein M (2021). Designing provider-focused implementation trials with purpose and intent: introducing the PRECIS-2-PS tool. Implement Sci.

[23] Gaglio B, Phillips SM, Heurtin-Roberts S, Sanchez MA, Glasgow RE (2014). How pragmatic is it? Lessons learned using PRECIS and RE-AIM for determining pragmatic characteristics of research. Implement Sci.

[24] Johnson KE, Neta G, Dember LM, Coronado GD, Suls J, Chambers DA (2016). Use of PRECIS ratings in the National Institutes of Health (NIH) health care systems research collaboratory. Trials.

[25] Glasgow RE, Gaglio B, Bennett G, Jerome GJ, Yeh HC, Sarwer DB (2012). Applying the PRECIS criteria to describe three effectiveness trials of weight loss in obese patients with comorbid conditions. Health Serv Res.

[26] Palmer JA, Mor V, Volandes AE, McCreedy E, Loomer L, Carter P (2018). A dynamic application of PRECIS-2 to evaluate implementation in a pragmatic, cluster randomized clinical trial in two nursing home systems. Trials.

[27] Zatzick D, Donovan DM, Jurkovich G, Gentilello L, Dunn C, Russo J (2014). Disseminating alcohol screening and brief intervention at trauma centers: A policy-relevant cluster randomized effectiveness trial. Addiction (Abingdon, England).

[28] Zatzick D, Jurkovich G, Heagerty P, Russo J, Darnell D, Parker L (2021). Stepped collaborative care targeting posttraumatic stress disorder symptoms and comorbidity for US trauma care systems: a randomized clinical trial. JAMA Surg.

[29] Zatzick D, Russo J, Darnell D, Chambers DA, Palinkas L, Van Eaton E (2016). An effectiveness-implementation hybrid trial study protocol targeting posttraumatic stress disorder and comorbidity. Implement Sci.

[30] Zatzick D, Donovan DM, Dunn C, Jurkovich GJ, Wang J, Russo J (2013). Disseminating Organizational Screening and Brief Intervention Services (DO-SBIS) for alcohol at trauma centers study design. Gen Hosp Psychiat.

[31] American College of Surgeons Committee on Trauma. Resources for Optimal Care of the Injured Patient; 2020.

[32] American College of Surgeons Committee on Trauma (2014). Resources for optimal care of the injured patient: 2014.

[33] American College of Surgeons Committee on Trauma. Resources for optimal care of the injured patient. Chicago: American College of Surgeons; 2006.

[34] Wagner AW, Zatzick DF, Ghesquiere A, Jurkovich GJ (2007). Behavioral activation as an early intervention for posttraumatic stress disorder and depression among physically injured trauma survivors. Cogn Behav Pract.

[35] March J, Silva S, Petrycki S, Curry J, Wells K, Fairbank J (2004). Fluoxetine, cognitive-behavioral therapy, and their combination for adolescents with depression: Treatment for Adolescents With Depression Study (TADS) randomized controlled trial. JAMA.

[36] Kratochvil CJ, Simons A, Vitiello B, Walkup J, Emslie G, Rosenberg D (2005). A multisite psychotherapy and medication trial for depressed adolescents: Background and benefits. Cognitive Behav Pract.

[37] Reidbord SP, Redington DJ (1993). Nonlinear analysis of autonomic responses in a therapist during psychotherapy. J Nerv Ment Dis.

[38] Palinkas LA, Whiteside L, Nehra D, Engstrom A, Taylor M, Moloney K (2020). Rapid ethnographic assessment of the COVID-19 pandemic April 2020 ‘surge’ and its impact on service delivery in an acute care medical emergency department and trauma center. BMJ Open..

[39] Palinkas LA, Engstrom A, Whiteside L, Moloney K, Zatzick D (2021). A rapid ethnographic assessment of the impact of the COVID-19 Pandemic on mental health services delivery in an acute care medical emergency department and trauma center. Adm Policy Ment Health.

[40] Miller WL, Crabtree BF. Primary care research: A multimethod typology and qualitative road map. Doing qualitative research. Research methods for primary care, Vol. 3. Thousand Oaks: Sage Publications, Inc; 1992. p. 3–28.

[41] Saldaña J (2016). The coding manual for qualitative researchers.

[42] Koo TK, Li MY (2016). A guideline of selecting and reporting intraclass correlation coefficients for reliability research. J Chr Med.

[43] Cicchetti DV (1994). Guidelines, criteria, and rules of thumb for evaluating normed and standardized assessment instruments in psychology. Psychol Assess.

[44] Powell BJ, Waltz TJ, Chinman MJ, Damschroder LJ, Smith JL, Matthieu MM (2015). A refined compilation of implementation strategies: results from the Expert Recommendations for Implementing Change (ERIC) project. Implement Sci.

[45] Designing with Implementation and Dissemintation in Mind: Introduction. In: Rethinking Clinical Trials: A Living Textbook of Pragmatic Clinical Trials. Bethesda: NIH Health Care Systems Research Collaboratory. Available from: https://rethinkingclinicaltrials.org/welcome-to-rethinking-clinical-trials/. Accessed 6/1/22.

[46] Huang SS, Septimus E, Kleinman K, Moody J, Hickok J, Avery TR (2013). Targeted versus universal decolonization to prevent ICU infection. N Engl J Med.

[47] Septimus E, Hickok J, Moody J, Kleinman K, Avery TR, Huang SS (2016). Closing the translation gap: toolkit-based implementation of universal decolonization in adult intensive care units reduces central line–associated bloodstream infections in 95 community hospitals. Clin Infect Dis.

[48] Pocobelli G, Yu O, Fuller S, Fraser JR, Wartko PD, Chen L (2018). One-step approach to identifying gestational diabetes mellitus: association with perinatal outcomes. Obstet Gynecol.

[49] Crable EL, Lengnick-Hall R, Stadnick NA, Moullin JC, Aarons GA (2022). Where is "policy" in dissemination and implementation science? Recommendations to advance theories, models, and frameworks: EPIS as a case example. Implement Sci.

[50] Rabin BA, McCreight M, Battaglia C, Ayele R, Burke RE, Hess PL (2018). Systematic, multimethod assessment of adaptations across four diverse health systems interventions. Front Public Health.

[51] DeBar L, Benes L, Bonifay A, Deyo RA, Elder CR, Keefe FJ (2018). Interdisciplinary team-based care for patients with chronic pain on long-term opioid treatment in primary care (PPACT) - Protocol for a pragmatic cluster randomized trial. Contemp Clin Trials.

